# To a Cab of the Night

**Published:** 1910-06

**Authors:** John E. Rosser


### To a Cab of the Night.

BY JOHN E. ROSSER.

Ebon pander to the Lower Venus,
Stealthy vampire of the night,
Sooty Ganymede of bloat Silenus,
Leering with your lech'rous light,
Sibilantly like the hissing adder,
Mumbling like a toothless hag,
Swift you steal to make a life sadder,
Virtue in the slime to drag;
Demon-eyed, you haunt the harlot's highway,
Rendezvous of blazoned shame,
Secret loves you aid on hidden byway,
Serving here your chiefest aim;
Yours to blight the sacred home-ties,
Yours to damn, debauch and blight,
Yours to heap full the cup of miseries,
I loathe you, Cab of the Night!

